# New perspectives in biological crystallography

**DOI:** 10.1107/S205225251400373X

**Published:** 2014-02-28

**Authors:** Edward N. Baker

**Affiliations:** a School of Biological Sciences, University of Auckland, Private Bag 92019, Auckland, New Zealand

**Keywords:** biological crystallography, editorial, IUCrJ

## Abstract

Biological crystallography has never been more vibrant. Celebrating this, **IUCrJ** invites high-impact papers from across the whole spectrum of structural biology and medicine.

This year, the International Year of Crystallography, is a momentous one for the whole crystallographic community, celebrating 100 years since the birth of X-ray crystallography. It also sees the launch of a new open-access journal from the IUCr, **IUCrJ**. What does this mean for Biology and Medicine, two of the many disciplines that will be represented in **IUCrJ**?

When *Acta Crystallographica* was launched in 1948, at the time of the founding of the International Union of Crystallography, biological crystallography was in its infancy. Not in terms of thinking, because visionary crystallographers such as J. D. Bernal and Dorothy Hodgkin could already see what needed to be done, and most of today’s methods already existed as concepts. What was lacking was the technology. The discovery of the structure of DNA in 1953 and the first protein structure, myoglobin in 1958, announced the arrival of X-ray crystallography as the premier method for biological structure analysis, and Nobel Prizes for Crick, Watson and Wilkins, and Kendrew and Perutz came in 1962.

Since then we have experienced an extraordinary expansion of crystallography into biology and medicine, driven by technological advances – recombinant DNA technology, computing, synchrotrons, improved crystallization methods, new phasing and refinement algorithms *etc*. On the one hand there have been spectacular achievements in defining the machinery of life; the ribosome, GPCRs and other membrane proteins, DNA replication, the immune system, whole viruses. On the other hand, crystallography has become integral to modern drug discovery for its ability to define, in atomic detail, how small molecules bind to proteins. We understand the chemistry of biology so much better.

There is an inevitable evolution in science in which old approaches become superseded by new ones and are often discarded and forgotten. This has not happened with crystallography. The same fundamental principles are still in place and relevant, 100 years on; we have just learned to apply them in different ways. We have also broadened our view of what is crystallography, to include different kinds of scattering (electrons, neutrons, X-rays) from different kinds of samples (liquids, amorphous and semi-crystalline materials, fibres, crystals). This goes right back to J. D. Bernal’s vision set out in Dorothy Hodgkin’s 1964 Nobel Lecture (see box[Chem scheme1]).
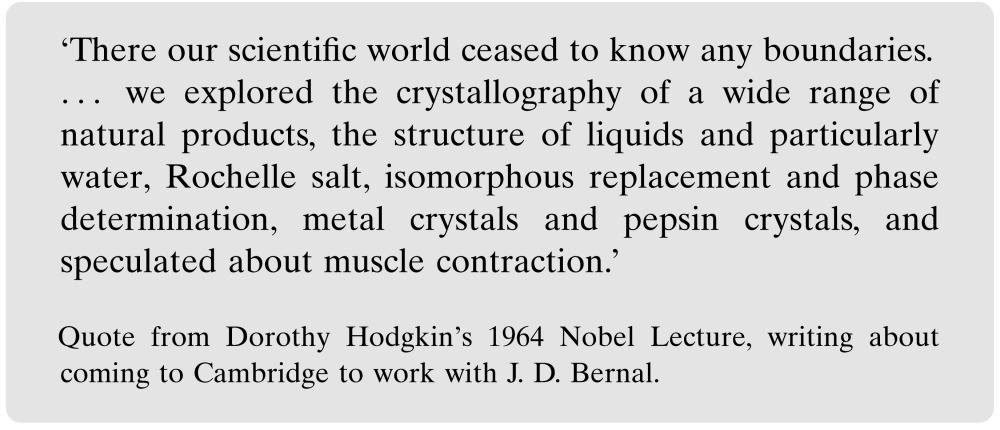


Today we stand on the cusp of new, exciting opportunities and crystallography has never seemed so vibrant. The advent of the X-ray free electron laser (XFEL) has opened up spectacular new possibilities, for example for using the tiniest of crystals, and concepts like serial femtosecond crystallography are becoming a reality. This is a field for the adventurous, and will make it possible to address biological systems that have so far been outside the range of conventional crystallography. On the other hand, conventional crystallography, with its power to define the chemistry of biological systems, will undoubtedly remain the bedrock of biological crystallography and its applications to medicine.

Just as crystallography has evolved, so has scientific publishing. Many journals are no longer published in print form, and researchers access the literature in different ways. A large number of biological and biomedical journals now publish the results of crystallographic studies, and the choice of where to publish can be a daunting one. It may be wishful thinking to imagine that impact factors will lose their current dominant role, but what matters in the end is whether one’s research is seen, and by whom.

For this reason the launch of **IUCrJ** as an open-access journal covering the full breadth of crystallography is an exciting and important one. Increasingly, today’s structural biologists must be multi-skilled, combining structural analyses with biological and biophysical studies, bioinformatics and computational studies. Structural analyses commonly involve combinations of techniques: high-resolution crystallography for parts of a system that can be crystallized, NMR to address dynamics, and small angle scattering or cryo-EM for the whole system. These are compelling reasons for publication in **IUCrJ**: high visibility of research that exploits the power of crystallography in its broadest sense, whether it involves focusing the highest possible resolution on a drug binding to a receptor, or exploiting a wider repertoire of approaches to address large, complex biological systems. We urge structural biologists to see this new IUCr journal as the natural home for innovative science across the whole spectrum of disciplines encompassed by the IUCr.

